# Case Report: Successful revascularization in massive pulmonary embolism with a large protruding thrombus and dilated cardiomyopathy

**DOI:** 10.12688/f1000research.28311.3

**Published:** 2021-03-22

**Authors:** Hendri Susilo, Rerdin Julario, Citrawati Dyah Kencono Wungu

**Affiliations:** 1 Department of Cardiology and Vascular Medicine, Airlangga University, Surabaya, Indonesia; 2 Department of Physiology and Medical Biochemistry, Airlangga University, Surabaya, Indonesia

**Keywords:** Massive pulmonary embolism, large protruding thrombus, unstable hemodynamic, reperfusion

## Abstract

Pulmonary embolism is a potentially life-threatening condition. Despite advances in diagnostics, lack of consensus and delays in determining the diagnosis of pulmonary embolism are still important problems. We report the diagnosis and management of a 37-year-old man suffering from massive pulmonary embolism, a large protruding thrombus, and dilated cardiomyopathy. Echocardiography showed dilatation of all cardiac chambers, a large protruding thrombus in the right atrium to the inferior vena cava, impaired left and right ventricular systolic function, and global hypokinetic of the left ventricle with eccentric left ventricular hypertrophy. A thoracic computerized tomography scan showed pulmonary embolism with infarction. The patient’s blood pressure was 60/40 mmHg and heart rate was 110 bpm. The patient was diagnosed with high-risk acute pulmonary embolism. We gave him hemodynamic support and reperfusion therapy with a loading dose of 250,000 units of Streptokinase followed by 100,000 units/hour for 24 hours. After revascularization, the patient's hemodynamic condition improved. The diagnosis of acute pulmonary embolism is based on clinical symptoms, hemodynamic changes, or radiological examination. Unstable hemodynamic underlies high-risk stratification. Hypotension or shock results from obstruction of the pulmonary artery which causes increased right ventricular afterload and acute right ventricular dysfunction. Reperfusion with thrombolysis therapy could provide good outcomes in this patient. Prolonged anticoagulation should be given to prevent the recurrence of venous thromboembolism.

## Introduction

Venous thromboembolism, a clinical presentation of deep vein thrombosis (DVT) or pulmonary embolism, is the third most commonly found acute cardiovascular syndrome after myocardial infarction and stroke ^1^. Pulmonary embolism is a potentially life-threatening condition. Most patients die from pulmonary embolism within the first few hours of the event. Despite advances in diagnostics, delay in determining pulmonary embolism diagnosis is still a significant problem ^2^.

 Pulmonary embolism contributes to approximately 300,000 deaths per year in the United States ^3^. This makes pulmonary embolism one of the high-rank causes of cardiovascular death. In six European countries, over one million venous thromboembolism events or deaths per annum occurred in 2004. Of these patients, 34% died suddenly or within a few hours of the acute event, before therapy could be administered ^4^. Clinicians should be able to better recognize the signs and symptoms of acute pulmonary embolism; thus, diagnosis and management can be determined quickly and accurately to reduce patient’s mortality. We present a rare case of protruded thrombus in the right atrium passing through the tricuspid valve which position moving from the inferior vena cava towards the right atrium, causing a massive pulmonary embolism and dilated cardiomyopathy. We successfully performed revascularization in such case.

## Case presentation

A 37-year-old Indonesian man was referred to the emergency department of Dr. Soetomo General Hospital, Indonesia, in June 2019, with complaints of shortness of breath and swollen legs. His occupation was a farmer. Two months earlier, the patient went to the public health center for a prolonged cough (± five months), which was sometimes accompanied by shortness of breath. In the public health center, acid-fast bacillus (AFB) testing was performed with a negative result. Based on a chest radiograph, the doctor decided to give group 1 anti-tuberculosis drugs through the public health center. The treatment prescribed was fixed-dose combination, four tablets daily taken orally, with the composition of each tablet as follows: Rifampicin 150 mg, Isoniazid 75 mg, Pirazinamide 400 mg, and Ethambutol HCl 75 mg. Thus, in the emergency department, he was referred to the pulmonology department with a diagnosis of pulmonary tuberculosis.

In addition, the patient also had a history of proximal bilateral femoral deep vein thrombosis (DVT) of the left inferior limb proven by ultrasonography examination; the patient had undergone a thrombectomy a month before his referral to the hospital. The complaints of swollen leg were slightly reduced at that time; however, had recurred again, accompanied by swelling on his right limb.

The patient had been diagnosed with uncontrolled diabetes mellitus and heart failure one month before admission. He received subcutaneous injection 8 units of insulin aspart three times a day before meals, captopril 6.25 mg every eight hours orally, spironolactone 25 mg once daily orally, digoxin 0.25 mg once daily orally, codeine 10mg every eight hours orally, and rivaroxaban 15 mg every twelve hours orally.

When admitted to the pulmonary ward, the patient complained of shortness of breath, accompanied by pain and swelling in both legs. His blood pressure was 120/80 mmHg, pulse 128 beats per minute, respiratory rate 26 breaths per minute, and 3 liters per minute oxygen via nasal cannula. Physical examination revealed jugular venous distention, bilateral basal rales, hepatomegaly, and pitting edema in lower extremities.

**Figure 1.**
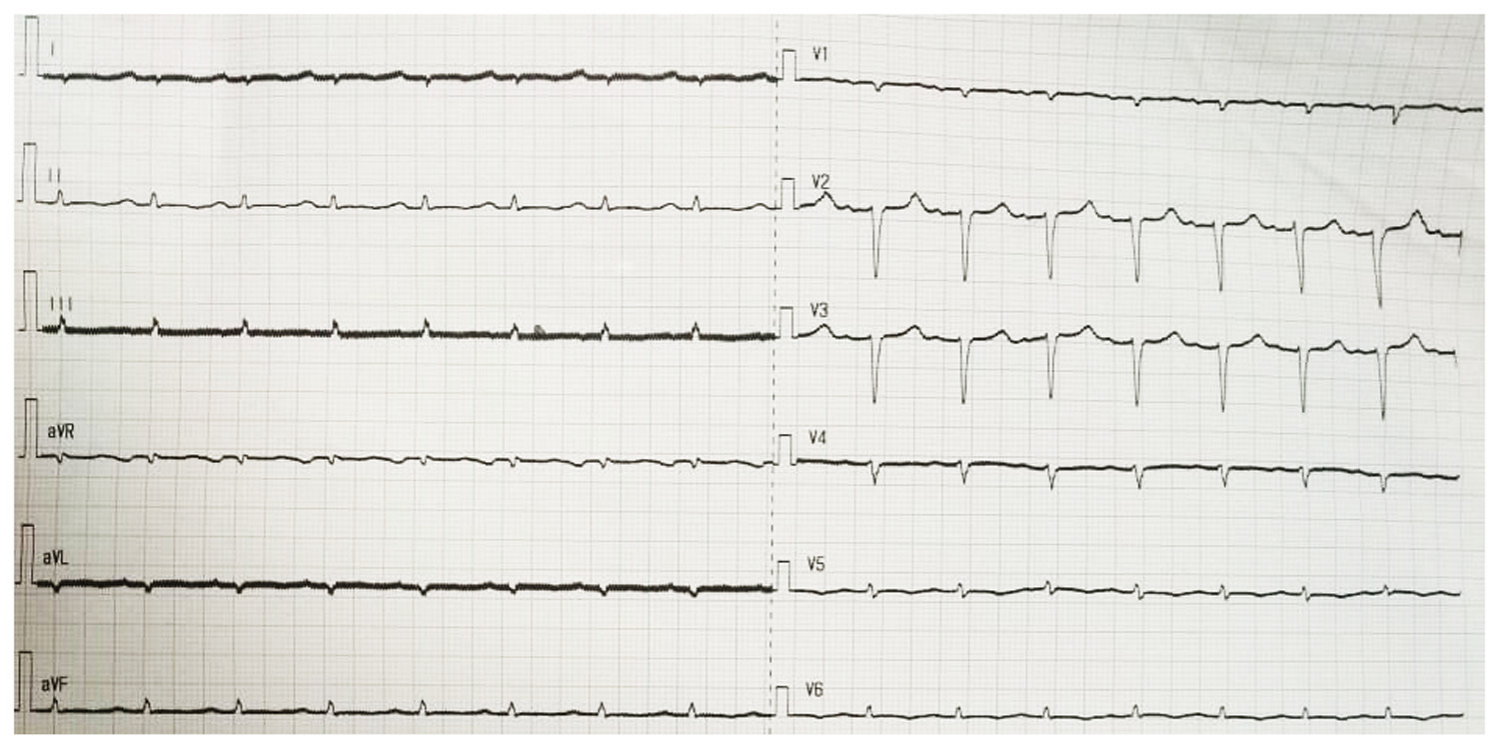
Electrocardiogram (ECG) of the patient.

**Figure 2.**
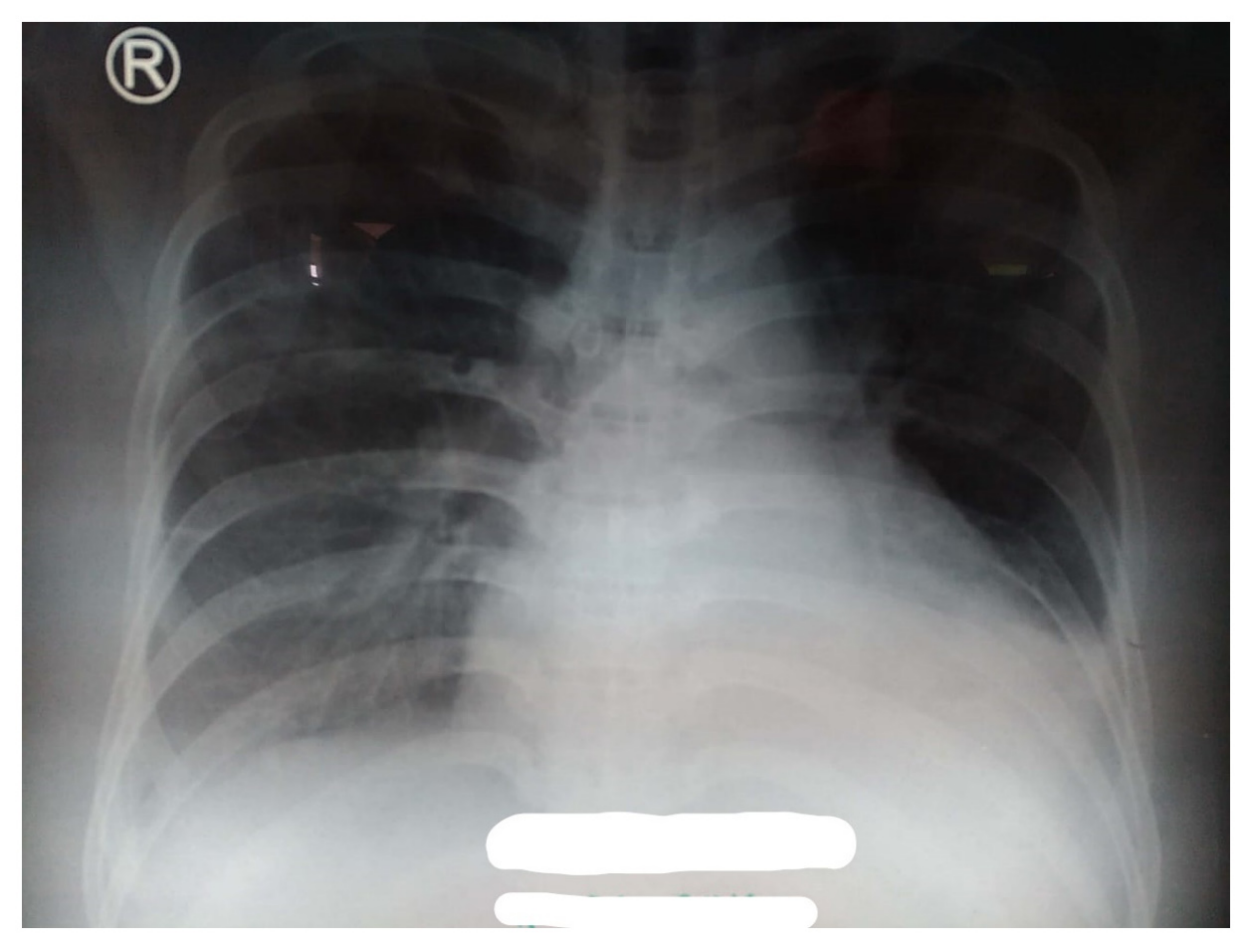
Chest X-ray of the patient.

**Figure 3.**
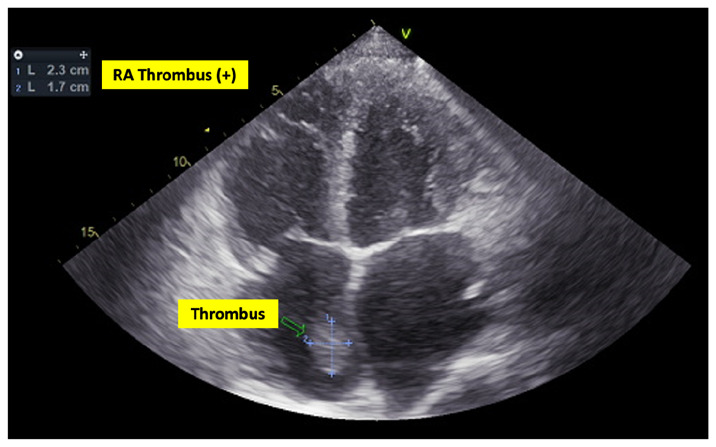
Transthoracic echocardiogram (TTE) showing thrombus in the right atrium. RA = right atrium.

**Figure 4.**
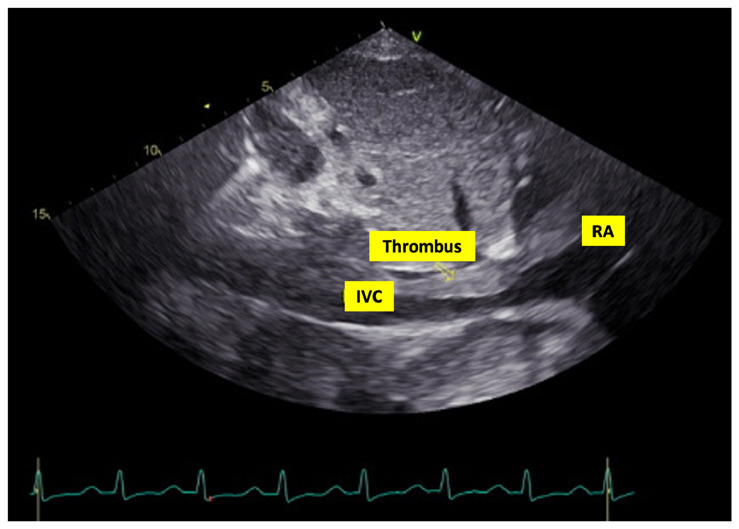
Transthoracic echocardiogram (TTE) showing thrombus in the inferior vena cava. RA = right atrium; IVC = inferior vena cava.

In laboratory findings, serum electrolytes revealed hypokalemia (K: 3.3 mmol/L; normal range 3.5–5.1 mmol/L), serum protein showed hypoalbuminemia (albumin: 3.1g/dL; normal range 3.4–5.0 g/dL), while other parameters were between normal limits. An electrocardiogram (ECG) showed sinus tachycardia rhythm 125 beats per minute, right-sided frontal axis, horizontal axis clockwise rotation, and slow progression of R waves at V1–V4 ( Figure 1). A chest X-ray showed cardiomegaly, pulmonary congestion, and minimal bilateral pleural effusion ( Figure 2). Echocardiographic examination revealed moderate mitral regurgitation (dilated mitral annulus), dilatation of all cardiac chambers (Left ventricular internal diastolic diameter 5.8 cm), visible thrombus in inferior vena cava to right atrium, decreased left and right ventricular systolic function (EF teich 35%, TAPSE 1.3 cm), and global hypokinetic of the left ventricle with eccentric left ventricular hypertrophy. The scans from a transthoracic echocardiogram (TTE) showing thrombus is shown in Figure 3 and Figure 4.

**Figure 5.**
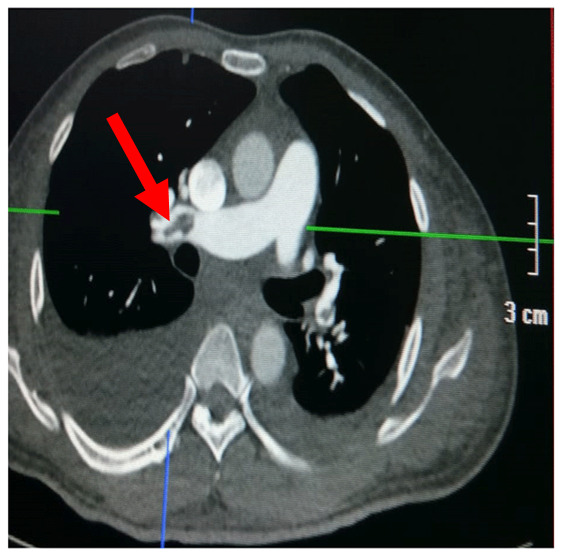
Chest CT scan of the patient. Red arrow showing the location of the thrombus.

**Figure 6.**
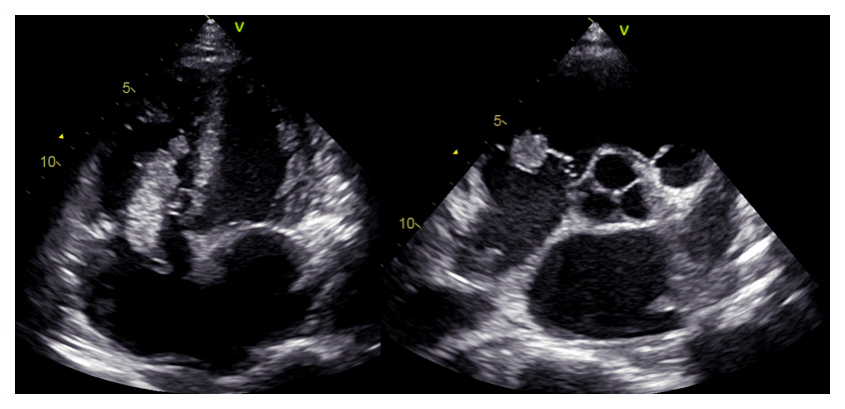
Transthoracic echocardiogram (TTE) showing protruded thrombus in the right atrium passes through the tricuspid valve.

**Figure 7.**
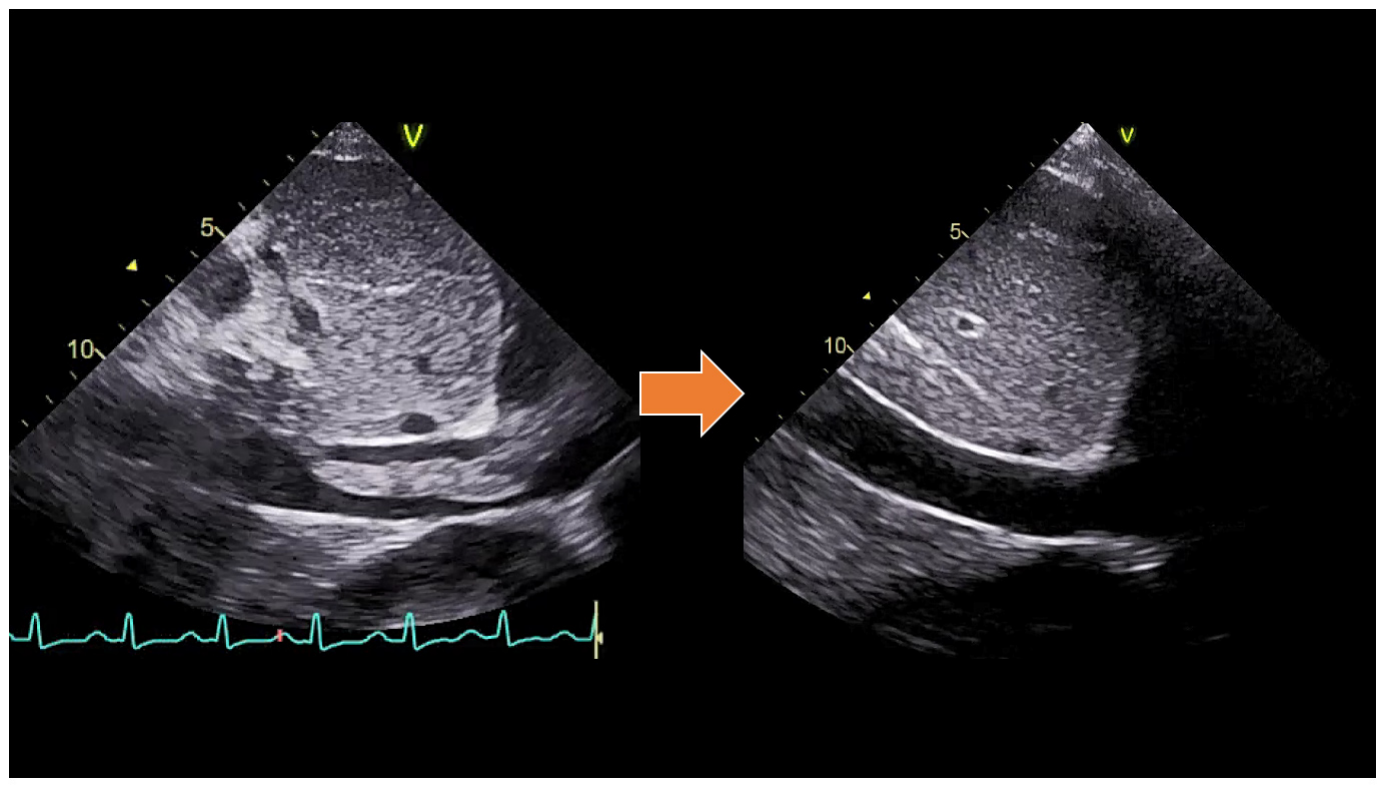
Transthoracic echocardiogram (TTE) comparison showing the position of the thrombus moving from the inferior vena cava towards the right atrium.

A chest CT scan ( Figure 5) showed right pulmonary artery embolism at ± 5.9 cm from bifurcation on the anterior side of the intermediate right bronchus; emboli on the left pulmonary artery bifurcation and the left pulmonary artery basal part; multiple right intraatrial hypodense lesions not showing contrast enhancement leading to a visualization of the right intraatrial thrombus; pulmonary infarction in the lateral-posterior segment of the base of the inferior lobe of the right lung, the lateral-posterior segment of the base of the inferior lobe of the left lung, and the anterior segment of the superior lobe of the left lung; and superior vena cava thrombus at VTH level 1-5. Figure 6 shows the protruded thrombus in the right atrium passing through the tricuspid valve. TTE also showed the position of the thrombus moving from the inferior vena ca va towards the right atrium ( Figure 7). The movement of the large protruding thrombus can be seen in the supplementary video files 1–3 ^5– 7^.

In the course of the assessments, no clinical, laboratory, or radiological signs of pulmonary tuberculosis were found. Eventually, the patient was transferred to the cardiology ward with the assessment of dilated cardiomyopathy + acute decompensated heart failure + deep vein thrombosis of the right and left inferior limbs + right atrial thrombus + pulmonary embolism + type II diabetes mellitus. During three days of treatment, the patient received Spironolactone 50 mg, Ramipril 5 mg, and low-dose Bisoprolol started at 1.25 mg each once daily orally, 20 mg of Furosemide by the intravenous injection every eight hours, subcutaneous injection of Enoxaparin 60 mg every twelve hours, and subcutaneous injection 6 units of insulin aspart three times daily before meals. However, in the course of this treatment in the cardiology ward, the patient suddenly complained of shortness of breath accompanied by chest pain and cold sweat. His blood pressure became 60/40 mmHg, pulse 110 beats per minute, and respiratory rate 28–30 breaths per minute, thus showing hemodynamic instability and shock. Therefore, he was reassessed as having high risk acute pulmonary embolism, and the patient was transferred to the cardiovascular care unit (CVCU) for observation and reperfusion therapy.

In the CVCU, we gave the patient hemodynamic support with Norepinephrine starting at 50 nanograms/kg/minute by titration. Reperfusion was carried out by giving a loading dose of 250,000 units of Streptokinase intravenously for 30 minutes, followed by 100,000 units of Streptokinase per hour for 24 hours with continuous intravenous pump. After revascularization, the patient's hemodynamic condition improved until vasopressors/inotropic drugs could be tapered off. TTE also showed the disappearance of the large protruding thrombus (supplementary video files 4–5) ^8, 9^. After the patient’s condition was stable, he was transferred to the cardiology ward until the patient was discharged after one week of thrombolytic treatment. In his discharge, rivaroxaban was prescribed at a daily dose of 20 mg as an oral anticoagulant for at least three months. The diabetes mellitus was well-controled with subcutaneous injection 6 units of insulin aspart three times daily before meals.

One week after discharge, the patient made a follow-up visit at the cardiology outpatient clinic. At that time, it was found that the patient's symptoms and exercise tolerance had improved, and his shortness of breath and swollen leg were reduced. The patient’s adherence to treatment was good, and there was neither sign of minor nor major bleeding due to anticoagulants’ use. Even though the clinical condition has improved, there was no improvement in the ejection fraction. Therefore, anticoagulant therapy and insulin injection were continued, accompanied by therapy for heart failure according to guideline-directed medical therapy (GDMT) for heart failure with reduced ejection fraction (HFrEF).

## Discussion

Pulmonary embolism is a critical medical emergency. It can lead to rapid deterioration of hemodynamic conditions with high mortality rate ^10^. Pulmonary embolism usually arises from thrombus originating from the deep venous system in the inferior limb. After heading to the lungs, a large protruding thrombus can attach to the branching of the main pulmonary artery or lobar branches, causing massive pulmonary embolism and hemodynamic disorders. This case report is unique as it showed massive pulmonary embolism due to a large protruding right-sided heart burden thrombus, which is rarely found in normal cases. This condition led to hemodynamic instability of the patient. This case also showed how immediate thrombolytic therapy could make the large thrombus disappear and provide a better outcome in such a patient. Pulmonary thromboembolism is not a basic disease in itself; instead, it is a complication of the underlying venous thrombosis ^11^. On vascular ultrasound examination in the present case, thrombi were found in the common femoral vein as well as the right and left popliteal veins. From the echocardiographic examination, thrombi in the inferior vena cava and the right atrium was also found. The limitation of this case report was the unexplained etiology of hypercoagulability of this patient.

### Clinical symptoms

Pulmonary embolism presentations are pleuritic chest pain, sudden onset, shortness of breath, and hypoxia. However, most patients with pulmonary embolism have no apparent symptoms. Conversely, symptoms can vary from sudden hemodynamic collapse to progressive shortness of breath. A diagnosis of pulmonary embolism should be suspected in patients with respiratory symptoms that cannot be explained by other alternative diagnoses ^12^.

One of these hemodynamic instability manifestations indicates a high-risk acute pulmonary embolism ^13^: first is cardiac arrest; second is an obstructive shock; and third is persistent hypotension. In this patient, clinical symptoms included shortness of breath, tachycardia, chest pain, coughing, signs of the inferior extremity DVT, tachypnoea, bilateral basal rhonchi, neck venous distention, minimal bilateral pleural effusion, and hemodynamic disorders. This patient had an obstructive shock; therefore, he was classified into a high-risk acute pulmonary embolism.

### Management

All patients with pulmonary embolism need immediate risk stratification. Thrombolytic therapy should be given to patients with acute pulmonary embolism with clinical hypotension (systolic pressure <90 mm HG) who do not have a high risk of bleeding. Thrombolysis in these patients should not be delayed because of the potential for irreversible cardiogenic shock. Thrombolytic therapy is recommended in certain patients with acute pulmonary embolism indicating a high risk of hypotension at initial clinical presentation or after starting anticoagulants Assessing the severity of pulmonary embolism, prognosis, and bleeding risk determines whether thrombolytic therapy can be given. Thrombolytic therapy is not recommended for most patients with acute pulmonary embolism that is not associated with hypotension ^14^. Primary reperfusion therapy - in most cases systemic thrombolysis - is the treatment of choice for patients with high-risk pulmonary embolism (with hemodynamic instability). Surgical pulmonary embolectomy or percutaneous catheter-directed treatment is an alternative reperfusion option in patients with contraindications to thrombolysis ^13^.

Before conducting thrombolysis therapy, several absolute and relative contraindications must be considered. Absolute contraindications for thrombolysis are ^13^: the history of hemorrhagic stroke or stroke of unknown cause; ischemic stroke during the last six months; neoplasms of the central nervous system; major trauma, surgery or head injury in the past three weeks; bleeding diathesis; and active bleeding. While relative contraindications are: transient ischemic attacks in the last six months; the use of oral anticoagulants; pregnancy or the first week postpartum; non-compressible puncture sites; traumatic resuscitation; traumatic hypertension (systolic blood pressure > 180 mmHg); severe/advanced liver disease; infectious endocarditis; and active peptic ulcer.

Acute right heart failure with low cardiac output is the leading cause of death in patients with high-risk acute pulmonary embolism. Long-term anticoagulation is very important for preventing the recurrence of DVT or pulmonary embolism, as even if a patient has been administered anticoagulants, DVT and pulmonary embolism still often recur. Apixaban, dabigatran, rivaroxaban, and edoxaban are alternatives to warfarin as the prophylaxis and treatment of pulmonary embolism. Apixaban, edoxaban, and rivaroxaban inhibit factor Xa, while dabigatran is a direct inhibitor of thrombin ^15^.

All patients with pulmonary embolism must be given anticoagulants for more than three months. The use of novel oral anticoagulants (NOAC) is considered to have a lower risk of bleeding than vitamin K antagonists. However, treatment with NOAC still has risks. Phase III clinical trials in venous thromboembolic patients with extended therapy show that major bleeding rates are around 1% and clinically relevant non-major bleeding is around 6% ^16, 17^.

In our patient, after the diagnosis of acute pulmonary embolism had been established, thrombolysis was the first choice. Reperfusion was carried out by giving a loading dose of 250,000 units of Streptokinase intravenously for 30 minutes, then followed by 100,000 units of Streptokinase per hour for 24 hours. Hemodynamic support was performed by giving Norepinephrine from 50 nanograms/kg/minute by titration.

## Conclusion

We reported the case of a 37-year-old man with a massive pulmonary embolism. The distinctiveness of this case was the presence of a large protruded thrombus in the right atrium passing through the tricuspid valve which position moving from the inferior vena cava towards the right atrium, causing the condition of massive pulmonary embolism and dilated cardiomyopathy in the patient. The diagnosis of acute pulmonary embolism was based on clinical symptoms, hemodynamic changes, echocardiographic examination, and a chest CT scan. Unstable hemodynamic conditions classified this patient in the high-risk stratification. Hypotension or shock resulted from acute right ventricular dysfunction due to the obstruction of the pulmonary artery embolus, which caused an increase in right ventricular afterload. Inotropic agents or vasopressors with Norepinephrine were needed to improve the hemodynamic profile. Successful revascularization was performed by thrombolysis with Streptokinase, which gave good outcomes in this patient. In conclusion, early diagnosis, risk assessment, and prompt treatment are important to treat patients with massive pulmonary embolism due to a large protruding thrombus. Hemodynamic deterioration, such as hypotension and shock, should be monitored in patients with massive pulmonary embolism to reduce mortality. Reperfusion therapy should be administered soon for patients with high-risk pulmonary embolism after assessing indication and contraindication for thrombolytic treatment. The problem with the current management of pulmonary embolism is when to start reperfusion therapy in patients with intermediate-high risk. Further research is needed to determine the proper management and immediate prompt treatment in such patients.

## Data availability

All data underlying the results are available as part of the article and no additional source data are required.

Movement of the large protruding thrombus (1) Transthoracic echocardiogram (TTE) showing movement of the protruding thrombus from the inferior vena cava towards the right atrium. Click here for additional data file. Copyright: © 2021 Susilo H et al. 2021

Movement of the large protruding thrombus (2) Transthoracic echocardiogram (TTE) showing movement of the protruding thrombus from the inferior vena cava towards the right atrium. Click here for additional data file. Copyright: © 2021 Susilo H et al. 2021

Movement of the large protruding thrombus (3) Transthoracic echocardiogram (TTE) showing movement of the protruding thrombus from the inferior vena cava towards the right atrium. Click here for additional data file. Copyright: © 2021 Susilo H et al. 2021

Disappearance of large protruding thrombus after revascularization (4) Transthoracic echocardiogram (TTE) showing the disappearance of the large protruding thrombus after revascularization. Click here for additional data file. Copyright: © 2021 Susilo H et al. 2021

Disappearance of large protruding thrombus after revascularization (5) Transthoracic echocardiogram (TTE) showing the disappearance of the large protruding thrombus after revascularization. Click here for additional data file. Copyright: © 2021 Susilo H et al. 2021

## Consent

Written informed consent for publication of their clinical details and clinical images were obtained from the patient.
