# Supplementary material for: Case Report: Successful revascularization in massive pulmonary embolism with a large protruding thrombus and dilated cardiomyopathy
Source: F1000Res. 2021 Mar 22;10:13. Originally published 2021 Jan 11. [Version 3] doi: 10.12688/f1000research.28311.3 (PMC7968529; doi:10.12688/f1000research.28311.3)
Supplement: Peer review history

# Peer review: Reviewer response for version 2

**DOI:** 10.5256/f1000research.54267.r81266

**Published:** 2021-03-16

**Authors:** Dyana Sarvasti

**Affiliations:** 1 Department of Internal Medicine, Faculty of Medicine, Widya Mandala Catholic University, Surabaya, Indonesia; 2 Husada Utama Hospital, Surabaya, Indonesia

The case reports submitted by the authors were fascinating and provided additional knowledge for clinicians. In terms of case descriptions and discussions that are presented, it is quite clear. However, the authors still lack detail in submitting their case reports. The figures included do not provide the reader with information. Likewise, writing words and sentences are sometimes confusing and mistyped. Even though this article only requires a minor revision, it is quite important to note. The following are proposed minor revisions to this article:

- The word “ **occured”** in “... *over one million VTE events or deaths per annum **occured** in 2004*…” should be “ **occurred**”.
- “ ***We presents** a rare case of protruded thrombus in the right atrium passing through...*” - It appears that the subject pronoun ***“We”*** and the verb ***“presents”*** are not in agreement. Consider changing the verb, which should be “ **We present**...”.
- *“...passing through the tricuspid valve, which **position** moving from the inferior vena cava towards the right atrium...” -* The plural verb ***“position”*** does not appear to agree with the singular subject ***"the tricuspid valve"***. Consider changing the verb form for the subject-verb agreement that should be “ **positions**”.
- “... *for a prolonged cough (± **5 **months)*...” - The numeral ***"5"*** is used instead of the word spelled out. Consider spelling out the number.
- It appears that **“ *anti tuberculosis*”** is missing a hyphen. Consider adding the hyphen(s).
- It appears that the **“ *fixed dose*”** is missing a hyphen. Consider adding the hyphen(s).
- *“... **4** tablets daily taken orally...” - *The numeral ***"4"*** is used instead of the word spelled out. Consider spelling out the number.
- *“...with **composition** of each tablet as follows:...” -* The noun phrase ***"composition"*** seems to be missing a determiner before it. Consider adding an article, **“the composition”**.
- The word ***“admited”***in *“When* ***admited*** *to the pulmonary ward...”* ****should be **“admitted”.**
- *“... **pulse 128 bpm, respiratory rate 26/minute, and 3 liter per minutes** oxygen via nasal cannula”*, should be **“pulse 128 beats per minute, respiratory rate 26 breaths per minute, and 3 liters per minute**”. The singular noun ***“liter”*** follows a number other than *one*. Consider changing the noun to the plural form. It seems that ***“minutes”*** may not agree in number with other words in this phrase.
- *“...hepatomegaly, and pitting edema in **both **lower extremities”*. It appears that ***“both”*** may be unnecessary in this sentence. Consider removing it.
- The noun phrase *“ **supplementary video”*** seems to be missing a determiner before it. Consider adding an article so that it reads, “ **the supplementary video**”.
- The noun phrase *" **intravenous injection"*** seems to be missing a determiner before it. Consider adding an article. “ **the intravenous injection**”.
- *“...Spironolactone 50 mg once daily **orally**, Ramipril 5 mg once daily **orally**, low-dose Bisoprolol started at 1.25 mg once daily **orally**...” -* It looks like your sentence contains a redundancy. Consider simplifying them.
- *“His blood pressure became 60/40 mmHg, heart rate 110 bpm, and respiratory rate 28–30/minute…” - *That **should be **“heart rate 110 beats per minute, and respiratory rate 28-30 breaths per minute”**.
- There is an inconsistency in using the word *" **pulse**"* or *" **heart rate**".* We recommend using one word that is uniform and consistent.
- *“...followed by 100,000 units of Streptokinase per hour for 24 hours with intravenous continuous pump...” - *It appears that the modifiers in the noun phrase *“ **intravenous continuous pump”*** are in the wrong order. Consider changing the word order to **“continuous intravenous pump”.**
- *“The diabetes mellitus was well- **controled** with subcutaneous injection of 6 units insulin aspart three times daily before meals.” -* The word *“* ***controled”***should be **“controlled”** and ***“6 units insulin” ***seems that preposition use may be incorrect here and should be **“6 units of insulin”.**
- The phrase ***“the use of anticoagulants”*** may be wordy. Consider changing the wording to be **“anticoagulants' use”**.
- The word ***“Eventhough”*** should be **“Even though”**.
- *“It can lead to rapid deterioration of hemodynamic **condition** with high...” -* It seems that *“ **condition”*** may not agree in number with other words in this phrase. It should be **“conditions"**.
- “ *From the echocardiographic examination, **the presence of** thrombi in the inferior vena cava and the right atrium was also found*.” - It appears that ***“the presence of”*** may be unnecessary in this sentence. Consider removing it.
- The phrase ***“presentations of pulmonary embolism”*** may be wordy. Consider changing the wording to **“pulmonary embolism presentations”**.
- The noun phrase ***"obstructive shock" ***seems to be missing a determiner before it. Consider adding an article so that it reads, **“an obstructive shock”.**
- *“...chest pain, coughing, signs of **inferior extremity**...” -* The noun phrase ***"inferior extremity”*** seems to be missing a determiner before it. Consider adding an article so that it reads, **“the inferior extremity”**.
- “ *This patient had an obstructive shock, **therefore**, he was classified into*...” - This sentence appears to consist of two independent clauses. Consider using a semicolon or period before **“therefore”.**
- ”... ***history*** *of hemorrhagic stroke or stroke of unknown cause*” - The noun phrase ***"history"*** seems to be missing a determiner before it. Consider adding an article so that it reads, **“the history”**.
- “ *Reperfusion was carried out by giving a loading dose of 250,000 units of Streptokinase **intraveneously** for 30 minutes*...” - It should be **“intravenously”**.
- *“We reported the case of a 37-year-old man with **massive** pulmonary embolism”*. It seems that article use may be incorrect here. It should be **“a massive”**.
- “ *The distinctiveness of this case was the **present **of a large protruded thrombus*...” - The word *“ **present”*** doesn’t seem to fit this context. Consider replacing it with a different one. It should be **“presence”**.
- “ *Further research is needed to determine the **right** management and immediate prompt treatment in such patients*” - It appears that the noun ***"management"*** might combine better with an adjective other than ***"right"*.** Consider rewriting this word pair or choosing a synonym for " **right"**. It should be **“proper”**.
- “ *Written informed consent for publication of their clinical details and clinical images **was **obtained from the patient*” - It seems that the verb **did** not agree with the subject. Consider changing the verb form. It should be **“were”**.
- It is best if all the abbreviations are written as their meanings (VTE, LVIDd, RA, IVC, LVH).
- Please clarify and tidy up the electrocardiogram image.
- Please write a description below the figures about the explanation of an abbreviation that has been written in the figure.
- Please correct the word ***"trombus"*** to **"thrombus"** in figure 3 and figure 4.

Are enough details provided of any physical examination and diagnostic tests, treatment given and outcomes?

Yes

Is the case presented with sufficient detail to be useful for other practitioners?

Yes

Is sufficient discussion included of the importance of the findings and their relevance to future understanding of disease processes, diagnosis or treatment?

Yes

Is the background of the case’s history and progression described in sufficient detail?

Yes

Reviewer Expertise:

Clinical cardiology, cardiovascular prevention and rehabilitation.

I confirm that I have read this submission and believe that I have an appropriate level of expertise to confirm that it is of an acceptable scientific standard.

# Response

**Published:** 2021-03-17

**Authors:** Citrawati Wungu

**Affiliations:** Airlangga University, Indonesia

We have repaired the grammatical errors, written the abbreviations as their meaning, corrected the word "thrombus" in the figures, and added the explanation of the abbreviations in the figures as suggested.

# Peer review: Reviewer response for version 2

**DOI:** 10.5256/f1000research.54267.r79576

**Published:** 2021-02-15

**Authors:** Henry Sutanto

**Affiliations:** 1 Department of Cardiology, Cardiovascular Research Institute Maastricht (CARIM), Maastricht, The Netherlands

Thanks for addressing my previous comments. I have no other questions or remarks, only that the manuscript writing and case reporting could still be improved.

Are enough details provided of any physical examination and diagnostic tests, treatment given and outcomes?

Partly

Is the case presented with sufficient detail to be useful for other practitioners?

Partly

Is sufficient discussion included of the importance of the findings and their relevance to future understanding of disease processes, diagnosis or treatment?

Partly

Is the background of the case’s history and progression described in sufficient detail?

Partly

Reviewer Expertise:

Cardiovascular disease, cardiogenetics, cardiac arrhythmias, arrhythmogenesis, inherited arrhythmia syndromes, computational modeling and bioinformatics.

I confirm that I have read this submission and believe that I have an appropriate level of expertise to confirm that it is of an acceptable scientific standard.

# Peer review: Reviewer response for version 1

**DOI:** 10.5256/f1000research.31314.r77083

**Published:** 2021-01-12

**Authors:** Henry Sutanto

**Affiliations:** 1 Department of Cardiology, Cardiovascular Research Institute Maastricht (CARIM), Maastricht, The Netherlands

In this case report, Dr. Susilo and colleagues reported a case of a man with pulmonary embolism (PE), right atrial (RA) thrombus and dilated cardiomyopathy (DCM). In general, the manuscript showed nicely the rapidly progressing PE into hemodynamic instability, which was immediately corrected following the administration of thrombolytic agent. However, I have some major and minor comments to be addressed by the authors, including the completeness of data.

- **Overall, the authors need to highlight the distinctiveness of this case, which is not well-defined in the current version of the manuscript.** It is currently unclear why this case is different than the other PE cases? The fact that one of the thrombi is located in the RA does help to stress the importance of this case report.
- **I would suggest the authors to arrange and discuss the data more systematically, and provide complete history and supporting data in the manuscript.** For example, the patient was diagnosed with diabetes mellitus and heart disease 1-month before admission. Which heart disease? What about the glycemic status prior to and after admission? Was the diabetes controllable?
- **The time course of clinical and laboratory parameters is lacking.** For example, it is currently unclear about the ejection fraction after discharge and during the follow-up visit? What about the hypercoagulable state and so on? These values are important to report in this manuscript.
- Here, the authors explicitly mentioned three observed phenomena in the title: 1) PE; 2) RA thrombus and 3) DCM. However, **the manuscript mainly focused on the PE and RA thrombus with a very limited discussion regarding DCM and its correlation with the other observed pathologies. **Please clarify the reason and adapt the discussion accordingly elaborating the potential interactions between those pathologies.
- In addition to the clinical presentations, please include the ECG recordings and other data justifying the diagnosis of acute PE during the shock event.
- The authors are requested to speculate on the origin of the RA thrombus. Is it primarily from the RA or is it a migrating thrombus? Was there any evidence of situations facilitating thrombus formation, such as hypercoagulability, atrial fibrillation, tricuspid valve disease, and so on prior to the incident? Provide some supporting data (e.g., lab results, etc.).
- The authors mentioned that the etiology of hypercoagulability in this patient is unknown. However, laboratory results showing hypercoagulability were not reported. Please add them into the study. What about the link between diabetes and hypercoagulable state, which has been discussed by many other publications? Please discuss this.
- Was left ventricular (LV) noncompaction observed in this patient? Elaborate your arguments.
- The rationale of DCM diagnosis in this patient is unclear. The authors need to provide a table containing checklist of clinical, imaging, and genetic data supporting this diagnosis. The authors can benefit from the paper by Mestroni et al. (Eur Heart J. 1999) ^1^ or Mathew et al. (Echo Res Pract. 2017) ^2^.
- Please confirm the nature of DCM in this patient? Familial or acquired? Include the family pedigree if there is an evidence of inherited DCM.
- Patients with DCM have a high risk of sudden cardiac death. Please explain the reason why in this patient, implantable cardioverter defibrillator (ICD) was not (planned to be) implanted? Moreover, the patient was hypokalemic, which poses to a bigger risk for life-threatening cardiac arrhythmias. Please also clarify the approach has been done to fix this problem.
- The authors mentioned in the abstract that echocardiography showed dilation of all cardiac chambers. However, no quantitative assessment of the atrial diameter provided. Please add.
- I am still not sure why anti-tuberculosis drugs were prescribed while AFB test was negative? Is there any involvement of these drugs on the clinical presentation of the patient?
- What is the rationale of administering rivaroxaban in the patient prior to the hospital admission? Was that due to the DVT? Please speculate on the reason why thrombi still persisted after rivaroxaban therapy (perhaps could also be supported by patient's lab results)?
- In Figure 5, please add some details / labels on the abnormalities observed in the CT scan, including the location of thrombi in the pulmonary artery.
- Avoid using numbers 1), 2), 3) in the text. Convert them into words: first, second, etc. if necessary.
- In the abstract: *“We gave him …”* please change into passive sentence similar to the rest of the manuscript.
- In the abstract: please add the administration route of streptokinase.
- Please convert the incidence of PE in six EU countries in the introduction into 1 in XXX. The way the authors reported the epidemiological data is not common.
- *“be better able to”* should be *“be able to better …”*
- *“… determined quickly and accurately to reduce **patients’** mortality”*
- *“the patient had **undergone** a thrombectomy a month before …”*
- *“SpO2 99% with 3 lpm nasal cannula” *should be *“3 liter per minutes oxygen via nasal cannula”.*
- *“moratality” should be “mortality”*

Are enough details provided of any physical examination and diagnostic tests, treatment given and outcomes?

Partly

Is the case presented with sufficient detail to be useful for other practitioners?

Partly

Is sufficient discussion included of the importance of the findings and their relevance to future understanding of disease processes, diagnosis or treatment?

Partly

Is the background of the case’s history and progression described in sufficient detail?

Partly

Reviewer Expertise:

Cardiovascular disease, cardiogenetics, cardiac arrhythmias, arrhythmogenesis, inherited arrhythmia syndromes, computational modeling and bioinformatics.

I confirm that I have read this submission and believe that I have an appropriate level of expertise to confirm that it is of an acceptable scientific standard, however I have significant reservations, as outlined above.

## References

[1] : Guidelines for the study of familial dilated cardiomyopathies. Collaborative Research Group of the European Human and Capital Mobility Project on Familial Dilated Cardiomyopathy. *Eur Heart J*.1999;20(2) : 10.1053/euhj.1998.1145 93-102 10.1053/euhj.1998.1145 10099905

[2] : Diagnosis and assessment of dilated cardiomyopathy: a guideline protocol from the British Society of Echocardiography. *Echo Res Pract*.2017;4(2) : 10.1530/ERP-16-0037 G1-G13 10.1530/ERP-16-0037 28592613PMC5574280

# Response

**Published:** 2021-01-27

**Authors:** Citrawati Wungu

**Affiliations:** Airlangga University, Indonesia

Thank you for your review. We have made revision according to your suggestions. These are our explanations regarding the comments:

1. We have added the distinctiveness of this case, that this was a rare case of protruded thrombus in the right atrium passing through the tricuspid valve which position moving from the inferior vena cava towards the right atrium, causing a massive pulmonary embolism and dilated cardiomyopathy.
2. We have added the history and supporting data as suggested.
3. We have added the explanation of the ejection fraction which had not improved at the follow-up visit. Ejection fraction was not an indicator for discharge in such patient. We have stated in the last paragraph of discussion section that the limitation of this case report was the unexplained etiology of hypercoagulability of this patient.
4. In this case report we did not focus on the DCM. However, DCM increases the risk of intracardiac thrombus which causes hypokinetic condition (venous stasis), a part of Virchow’s triad.
5. ECG in PE is less specific. The most common ECG finding in the setting of a pulmonary embolism is sinus tachycardia. S1Q3T3 is a specific pattern in PE, however, this pattern only occurs in about 10% of people with pulmonary embolisms and is similar to the ECG findings of a left posterior fascicular block, or LPFB (Mohammed and Elsayed, 2020).
6. It was a migrating thrombus from proximal femoral vein. The patient had a history of proximal bilateral femoral deep vein thrombosis (DVT) of the left inferior limb proven by ultrasonography examination and the patient had had a thrombectomy a month before his referral to the hospital. The complaints of swollen leg were slightly reduced at that time; however, had recurred again, accompanied by swelling on his right limb. He had no atrial fibrillation or tricuspid valve disease.
7. Endothelial abnormalities undoubtedly play a role in the enhanced activation of platelets and clotting factors seen in diabetes. Coagulation activation markers, such as prothrombin activation fragment 1+2 and thrombin-anti-thrombin complexes, are elevated in diabetes. The plasma levels of many clotting factors including fibrinogen, factor VII, factor VIII, factor XI, factor XII, kallikrein, and von Willebrand factor are elevated in diabetes. Conversely, the level of the anticoagulant protein C (PC) is decreased. The fibrinolytic system, the primary means of removing clots, is relatively inhibited in diabetes due to abnormal clot structures that are more resistant to degradation and an increase in plasminogen activator inhibitor type 1 (PAI-1). Increased circulating platelet aggregates, increased platelet aggregation in response to platelet agonists, increased platelet contractile force (PCF), and the presence of higher plasma levels of platelet release products, such as beta-thromboglobulin, platelet factor 4, and thromboxane B(2), demonstrate platelet hyperactivity in diabetes (Carr, 2001). However, our opinion is that the extensive thrombosis condition in this patient was not solely due to his diabetes.Hypercoagulability conditions can be related to genetic disorders, for example: Factor V Leiden (the most common), Prothrombin gene mutation, deficiencies of natural proteins that prevent clotting (such as antithrombin, protein C and protein S), elevated levels of homocysteine, elevated levels of fibrinogen or dysfunctional fibrinogen (dysfibrinogenemia), elevated levels of factor VIII (still being investigated as an inherited condition) and other factors including factor IX and XI, abnormal fibrinolytic system, including hypoplasminogenemia, dysplasminogenemia, and elevation in levels of plasminogen activator inhibitor (PAI-1) (Khan and Dickerman, 2006; Jan, 2020). However, we acknowledge that the weakness of this case report was that we did not have data for any genetic abnormalities.
8. There were no signs of LV noncompaction in this patient. No family history data was obtained. The Echo result did not show any trabeculations.
9. No genetic data available. DCM was diagnosed based on clinical and structural abnormalities by echocardiography. We found large ventricular enlargement with global hypokinetic and reduced ejection fraction.
10. ICD is indicated in heart failure patients with reduced EF (EF < 35) and no improvements with optimal medical treatment based on GDMT (Ponikowski *et al.*, 2016). In this patient we found high burden intracardiac thrombus, thus it will be so dangerous for us to perform ICD. It can make the thrombus escape dan migrate, thus we focus on the emergency and gave GDMT as the optimal treatment.
11. We have added atrial diameter as follows: LA mayor 6.4 cm, LA minor 47 cm; RA mayor 5.5 cm, RA minor 4.5 cm, RVDB 3.2 cm.
12. In the public health center, the doctor diagnosed the patient with clinical TB with negative AFB, however, in the course of the assessments in the hospital, no clinical, laboratory, or radiological signs of pulmonary tuberculosis were found, thus the patient was transferred to the cardiology ward.
13. Rivaroxaban was prescribed as a DVT therapy that has been known before by the referring doctor. Persistent thrombus may be due to the patient's hypercoagulability condition. However, we cannot give more specific conclusions regarding this matter due to limited data and laboratory examination results.
14. We have inserted the arrow to the figure. In manuscript we have stated that the CT scan showed right pulmonary artery embolism at ± 5.9 cm from bifurcation on the anterior side of the intermediate right bronchus.
15. We have made changes to the formatting as suggested.

**References:**

 Carr, M. E. (2001) ‘Diabetes mellitus: a hypercoagulable state.’, *Journal of diabetes and its complications*, 15(1), pp. 44–54. doi: 10.1016/s1056-8727(00)00132-x.

 Jan, B. S. P. T. A. G. A. (2020) *Hypercoagulability*. Treasure Island (FL): StatPearls Publishing. Available at: https://www.ncbi.nlm.nih.gov/books/NBK538251/.

 Khan, S. and Dickerman, J. D. (2006) ‘Hereditary thrombophilia’, *Thrombosis Journal*, 4, pp. 1–17. doi: 10.1186/1477-9560-4-15.

 Mohammed, Y. and Elsayed, H. (2020) ‘McGinn-White Sign or S1Q3T3-Pattern in Pulmonary Embolism ; Significance and Differential Diagnosis ; Narrative Updating Review’, *International Journal of Research Studies in Medical and Health Sciences*, 5(11), pp. 15–24. Available at: http://www.ijrsmhs.com/pdf/v5-i11/2.pdf.

 Ponikowski, P. *et al.* (2016) ‘2016 ESC Guidelines for the diagnosis and treatment of acute and chronic heart failure’, *European Heart Journal*, 37(27), pp. 2129-2200m. doi: 10.1093/eurheartj/ehw128.
